# Development and characterization of a new rat ocular hypertension model induced by intracameral injection of conjunctival fibroblasts

**DOI:** 10.1038/s41598-019-43048-2

**Published:** 2019-04-29

**Authors:** Ayumi Nakagawa, Osamu Sakai, Hideki Tokushige, Takashi Fujishiro, Makoto Aihara

**Affiliations:** 1 Central Research Laboratories, Senju Pharmaceutical Co., Ltd., 6-4-3, Minatojima-Minamimachi, Chuoku, Kobe, Hyogo, 650-0047 Japan; 2 Department of Ophthalmology, University of Tokyo, 7-3-1, Hongo, Bunkyoku, Tokyo, 113-8655 Japan

**Keywords:** Animal disease models, Disease model

## Abstract

Glaucoma is a chronic optic neuropathy that leads to visual field loss. Elucidating the mechanisms underlying glaucoma is essential for developing new treatments, such as neuroprotective drugs. Various glaucoma models based on the induction of intraocular pressure (IOP) elevation have been established for use in glaucoma studies. However, the time-dependent pathological changes accompanying IOP elevation have not been fully elucidated. In this study, rat conjunctival fibroblasts were injected into the anterior chamber of rat eyes, and IOP elevation was induced for 28 days. Glaucomatous signs such as optic nerve head cupping, retinal thinning, glial activation and apoptotic signaling in the retina were obvious in the cell-injected eyes on the 14th day after injection. The pattern of retinal ganglion cell (RGC) loss differed by the magnitude of IOP elevation. The number of RGCs decreased by 37.5% in eyes with IOP lower than 50 mmHg (Under-50) and by 88.0% in those with IOP higher than 50 mmHg (Over-50) 28 days after cell injection. The RGC counts were correlated with IOP in the Under-50 group but not in the Over-50 group. Our model may contribute to the investigation of pathogenic mechanisms of glaucoma and the development of new glaucoma treatments.

## Introduction

Glaucoma is a chronic degenerative optic neuropathy characterized by retinal ganglion cell (RGC) dysfunction, and it is a leading cause of visual field loss and irreversible blindness. Multiple and complex risk factors influence the development and progression of glaucoma. Elevated intraocular pressure (IOP) is one of the critical risk factors for glaucomatous optic neuropathy, and currently, the only evidence-based treatment for glaucoma is the lowering of IOP by pharmacological and surgical therapies^1,2^. IOP elevation causes optic nerve damage at the lamina cribrosa^3^, but the progression of optic nerve damage and the mechanism of RGC loss are not fully understood^4^. Thus, a chronic ocular hypertension (COH) model is essential for studying the pathophysiology of glaucoma^5,6^. Moreover, COH models are required for identifying therapeutic targets for not only IOP-lowering drugs but also neuroprotective drugs, as well as for the evaluation of neuroprotective drugs^7–9^

To date, various COH models have been established in monkeys^10–12^, rats^13^ and mice^14–17^ to investigate the molecular mechanisms underlying glaucomatous optic neuropathy progression. Monkey models of COH have particular advantages because the ocular anatomy is similar between monkeys and humans. However, preparing samples from a large number of monkeys is a challenging task. Although rodent models have some advantages over monkey models in experiments, some models require special devices or advanced techniques to induce IOP elevation. Therefore, establishing a new rodent model through a simple procedure would be helpful for the advancement of glaucoma research.

We previously reported a method for developing a COH model in ferrets^18^. In this model, conjunctival fibroblasts obtained from ferrets were injected into the anterior chamber of ferret eyes. The injected conjunctival fibroblasts accumulated at the angle of the anterior chamber and occluded the outflow, resulting in IOP elevation followed by an increase in optic nerve head (ONH) cupping and degeneration of axon in the optic nerve. This damage in the ferret COH model resembles the clinical pathogenesis of glaucoma. However, ferrets are difficult to handle and house. Nevertheless, ferrets are often used for visual-pathway investigations because they have binocular vision. Rats are easy to use in preclinical studies and have been extensively employed in glaucoma experiments^6^. In this study, we adapted the technique of intracameral fibroblast injection to rats and developed a simple model of COH that mirrored the pathology in human glaucoma.

Few studies have reported the time-dependent pathological changes, particularly the initial response, in glaucoma models. Thus, information regarding the time course of the neurological and morphological changes accompanying IOP elevation is limited. Therefore, we studied multiple pathological changes in our rat model at several time points. Furthermore, open angle glaucoma (OAG), which is the most common type of glaucoma, is characterized by long-term optic nerve neuropathy with a slow rate of progression. Animal models that display long-term IOP elevation but not abnormally high IOP are required to mimic the clinical pathology of OAG. However, the pathology in some experimental COH models differs from that in COH patients because those models are exposed to IOP values higher than 50 mmHg, leading to ischemic optic neuropathies such as acute primary angle closure (APAC)^19^. In this study, we also investigated the relationship between retinal morphological changes and the magnitude of IOP elevation and evaluated the pathological similarity between our model and glaucoma patients.

## Results

### Intraocular pressure (IOP)

**Figure 1:**
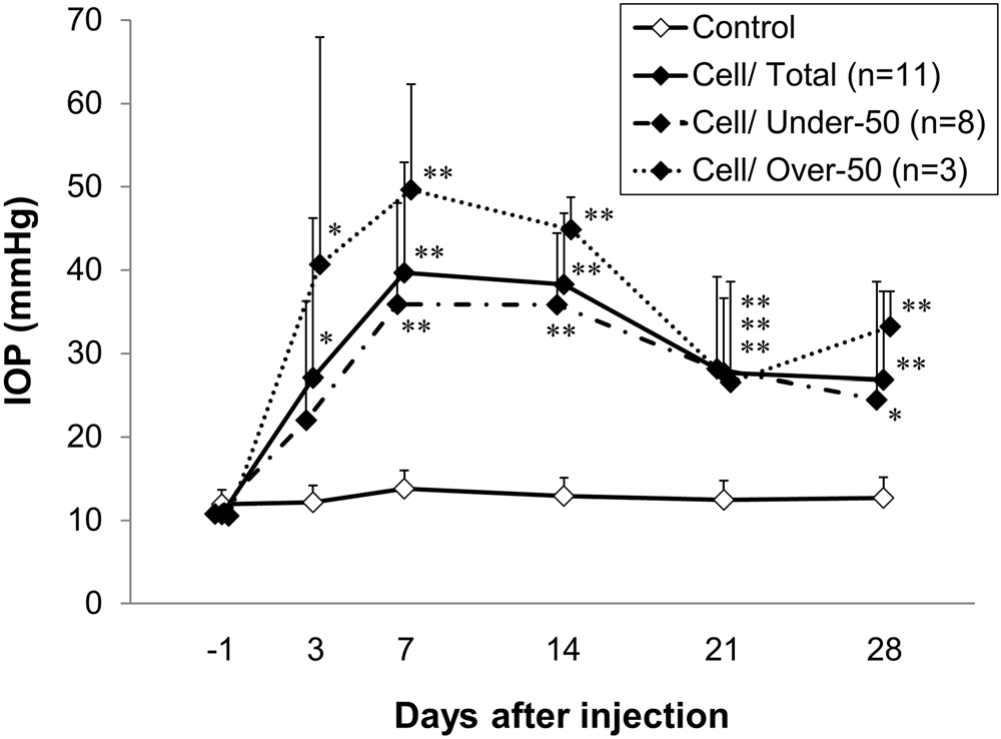
IOP profiles in eyes after cell injection and in control eyes. IOP elevation was first observed 3 days after cell injection and persisted for 28 days. The IOP values across all cell-injected eyes were 27.1 ± 19.1, 39.7 ± 13.3, 38.3 ± 8.5, 27.7 ± 8.9, and 26.9 ± 10.5 mmHg on the 3rd, 7th, 14th, 21st, and 28th day, respectively, after cell injection (n = 11). IOP was lower than 50 mmHg (Under-50) in 8 and higher than 50 mmHg (Over-50) in 3 of the 11 cell-injected eyes. Data are presented as the mean ± S.D. (mmHg). *p < 0.05, **p < 0.01 compared with the control (medium-injected) group (n = 8) (two-tailed Student’s *t*-test).

In this study, IOP was elevated in 39 of 55 eyes (70.9%) that were injected with conjunctival fibroblasts (i.e., the IOP difference between baseline and post-injection was higher than 10 mmHg) but not elevated (IOP differences consistently lower than 10 mmHg) in the remaining 16 injected eyes (29.1%). Animals without IOP elevation were excluded from the data analysis. Figure 1 shows IOP profiles for 28 days in the cell-injected eyes (n = 11) and in the medium-injected control eyes (n = 8). The mean IOP values were significantly higher in the cell-injected eyes than in the control (medium-injected) eyes 3 days after cell injection (27.1 ± 19.1 mmHg vs 12.2 ± 2.0 mmHg, p = 0.04, two-tailed Student’s *t*-test). The mean IOP values in the injected eyes reached its maximum value (39.7 ± 13.3 mmHg) 7 days after cell injection and was 26.9 ± 10.5 mmHg 28 days after injection. The IOP was lower than 50 mmHg in 8 of the 11 cell-injected eyes (the “Under-50” group: eyes consistently showing IOP lower than 50 mmHg throughout the measurement period) and higher than 50 mmHg in the remaining 3 eyes (the “Over-50” group: eyes showing IOP higher than 50 mmHg at least once during the measurement period). The mean IOP values in the Under-50 group were similar to those in the whole group of injected eyes. Conversely, the mean IOP values of eyes in the Over-50 group were 40.7 ± 27.3, 49.7 ± 12.7, 44.9 ± 3.9, 26.6 ± 12.0, and 33.2 ± 4.2 mmHg on the 3rd, 7th, 14th, 21st, and 28th day after cell injection, respectively. Overall, 11 eyes had an IOP higher than 50 mmHg on the 3rd or 7th day after cell injection, and it constituted 28.2% of the total number of eyes (39 eyes) with elevated IOP. An IOP higher than 50 mmHg may cause ischemic optic neuropathy, which differs from chronic glaucomatous optic neuropathy in OAG. Therefore, we compared the number of RGCs between the Over- and Under-50 groups using the following tests.

### Slit-lamp examination

**Figure 2:**
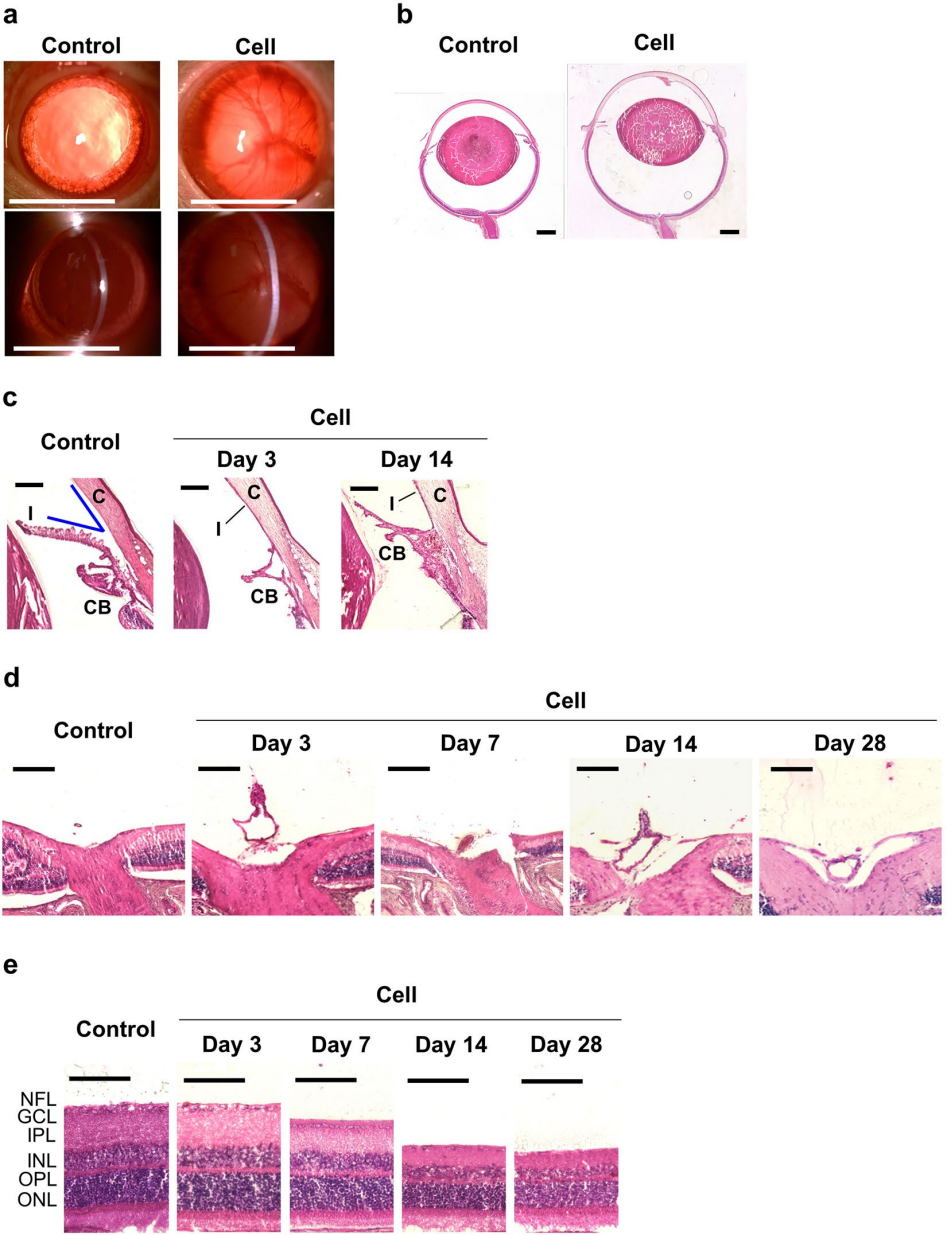
Histological analysis of the Under-50 group and the control group. Representative images of control eyes and Under-50 eyes on the 14th day (**a**,**b**), the 3rd and 14th day (**c**), or the 3rd, 7th, 14th, and 28th day after cell injection (**d**,**e**). (**a**) Slit-lamp examination of the anterior chamber of eyes under mydriasis. Cell accumulation in the anterior chamber and anterior synechiae was observed in the injected eyes from the 3rd to the 28th day after cell injection. Scale bars represent 5 mm. (**b**) Histological micrographs of hematoxylin and eosin (HE)-stained eyeballs. The cell-injected eyes gradually increased in size starting 7 days after the injection. Scale bars represent 1 mm. (**c**) Angle closure and (**d**) increase in optic nerve head cupping were observed 3 days after cell injection. Scale bars represent 200 µm. (**e**) Histological micrographs of retinal sections located 2 mm away from the optic nerve head. The retinal thickness in the Under-50 group decreased from 7 to 28 days after cell injection. Scale bars represent 100 µm. C, cornea; I, iris; CB, ciliary body; NFL, nerve fiber layer; GCL, ganglion cell layer; IPL, inner plexiform layer; INL, inner nuclear layer; OPL, outer plexiform layer; ONL, outer nuclear layer.

Injected cells accumulated on the corneal endothelium and anterior synechiae developed in eyes with elevated IOP. These observations were confirmed from 3 to 28 days after cell injection (Fig. 2a). There were no abnormal findings in the control (medium-injected) eyes.

### Histological analysis of eyeballs

To characterize the morphological changes after cell injection, we performed histological analysis on some eyes from the Under-50 group (n = 2–3). The size of the eyes with elevated IOP started to increase 7 days after cell injection (Fig. 2b). The angle was completely occluded due to the adhesion of the iris to the cornea (Fig. 2c) and ONH cupping was increased (Fig. 2d) in the eyes with elevated IOP beginning 3 days after cell injection. Moreover, the thicknesses of the nerve fiber layer (NFL) and the inner plexiform layer (IPL) decreased 7 days after cell injection, and thinning of the retina, including the inner nuclear layer (INL) and the outer nuclear layer (ONL), was observed 14 and 28 days after cell injection (Fig. 2e).

### Quantitative analysis of RGCs

**Figure 3:**
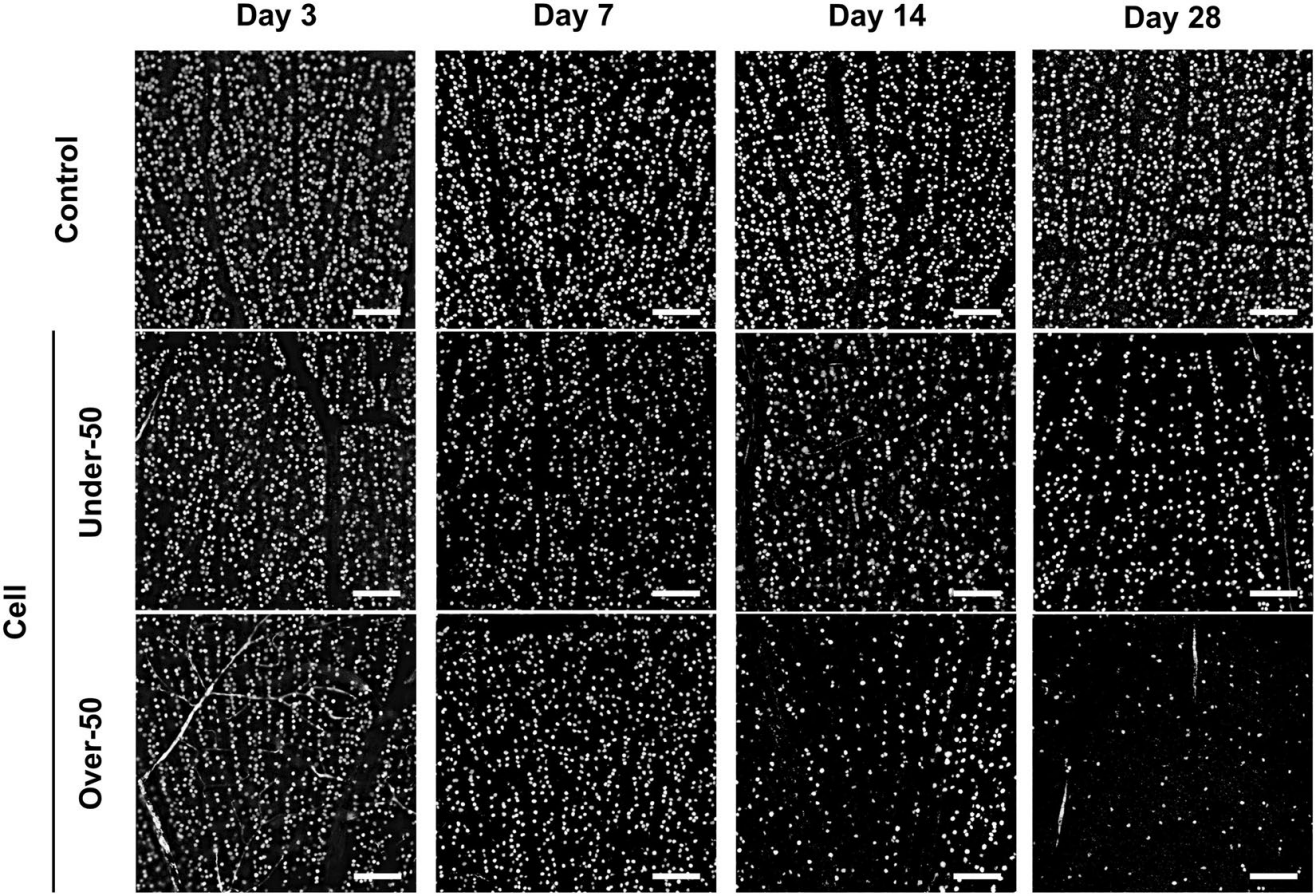
Representative micrographs of RGCs in retinal field. Representative micrographs showing RGCs located 1 mm away from the optic nerve head. BRN3A-positive RGCs were identified on flat-mounted retinae. The number of RGCs decreased in the injected eyes. In the Over-50 group, the numbers of RGCs were notably lower 14 and 28 days after cell injection than the numbers in the Under-50 group. Scale bars represent 100 µm.

**Figure 4:**
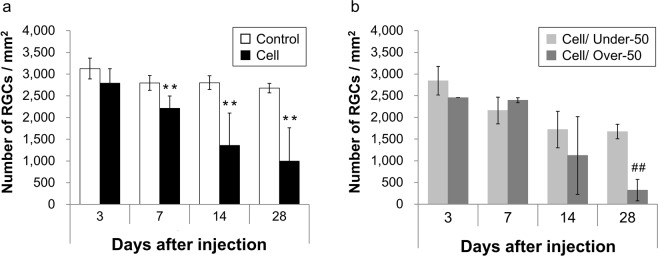
Quantitative analysis of RGCs. (**a**) Overall, the number of RGCs in the cell-injected eyes decreased by 10.8%, 21.0%, 51.4%, and 62.7% of that in the control group 3, 7, 14, and 28 days after cell injection, respectively. Data are presented as the mean ± S.D. (n = 5–9). **p < 0.01 compared with the control group (two-tailed Student’s *t*-test). (**b**) The number of RGCs in the Over-50 group was significantly decreased relative to that in the Under-50 group 28 days after cell injection. Data are presented as the mean ± S.D. (n = 1–7). ^##^p < 0.01 compared with the Under-50 group (two-tailed Student’s *t*-test).

BRN3A is specifically expressed in the nuclei of ganglion cells; thus, BRN3A-positive cells were counted as RGCs (Fig. 3). The number of BRN3A-positive cells decreased in a time-dependent manner in the Under-50 group, whereas in the Over-50 group, the number of BRN3A-positive cells markedly decreased from 14 days after cell injection onward. The overall mean number of RGCs on the 3rd, 7th, 14th, and 28th day after the induction of IOP elevation was 2790 ± 336, 2211 ± 286, 1361 ± 743, and 998 ± 764 cells/mm^2^ (n = 5–9), respectively, in all eyes with elevated IOP, and 3128 ± 240, 2798 ± 170, 2803 ± 160, and 2677 ± 110 cells/mm^2^ (n = 5–6), respectively, in control (medium-injected) eyes (Fig. 4a). The RGC counts were significantly lower in the injected eyes at 7, 14, and 28 days after cell injection than those in the control eyes (p = 0.0013, 0.0012, and 0.0009, respectively, two-tailed Student’s *t*-test). The rates of RGC loss on the 3rd, 7th, 14th, and 28th day after cell injection were 10.8%, 21.0%, 51.4%, and 62.7%, respectively. RGC counts at 3, 7, 14, and 28 days after cell injection were 2846 ± 330, 2158 ± 306, 1719 ± 419, and 1673 ± 166 cells/mm^2^ (n = 2–7) in the Under-50 group and 2455, 2395 ± 57, 1123 ± 896, and 323 ± 248 cells/mm^2^ (n = 1–3) in the Over-50 group, respectively (Fig. 4b). Thus, the RGC numbers were significantly lower in the Over-50 group than in the Under-50 group at 28 days after cell injection (p < 0.01, two-tailed Student’s *t*-test). The rates of RGC loss on the 3rd, 7th, 14th, and 28th days after cell injection were 9.0%, 22.9%, 38.7%, and 37.5% in the Under-50 group and 21.5%, 14.4%, 59.9%, and 88.0% in the Over-50 group, respectively.

### Correlation analysis between the number of RGCs and IOP change

**Figure 5:**
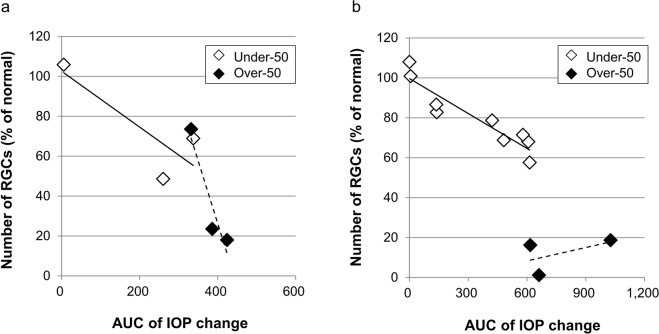
The correlation between RGC loss and area under the curve (AUC) of IOP change. The number of RGCs 14 days (**a**) and 28 days (**b**) after cell injection showed a negative correlation with the AUC of IOP change from baseline, except in the Over-50 group 28 days after cell injection.

RGC counts in the cell-injected eyes were compared to those in the contralateral (noninjected) normal eyes for each animal and expressed as a percentage of the RGC count of the cell-injected eyes. This percentage of RGCs showed a tendency toward a negative correlation with the area under the curve (AUC) of IOP change 14 days after cell injection (Under-50 group: n = 3, r = −0.8359, p = 0.3699; Over-50 group: n = 3, r = −0.9473, p = 0.2076) (Fig. 5a). On the 28th day after cell injection, there was an apparent negative correlation between the percentage of RGCs and the AUC of IOP in the Under-50 group (n = 9, r = −0.9308, p < 0.001) (Fig. 5b). In contrast, there was no negative correlation between the percentage of RGCs and the AUC of IOP in the Over-50 group (n = 3, r = 0.5278, p = 0.6460), and the number of RGCs showed an obvious reduction.

### TUNEL assay of retinal cells

**Figure 6:**
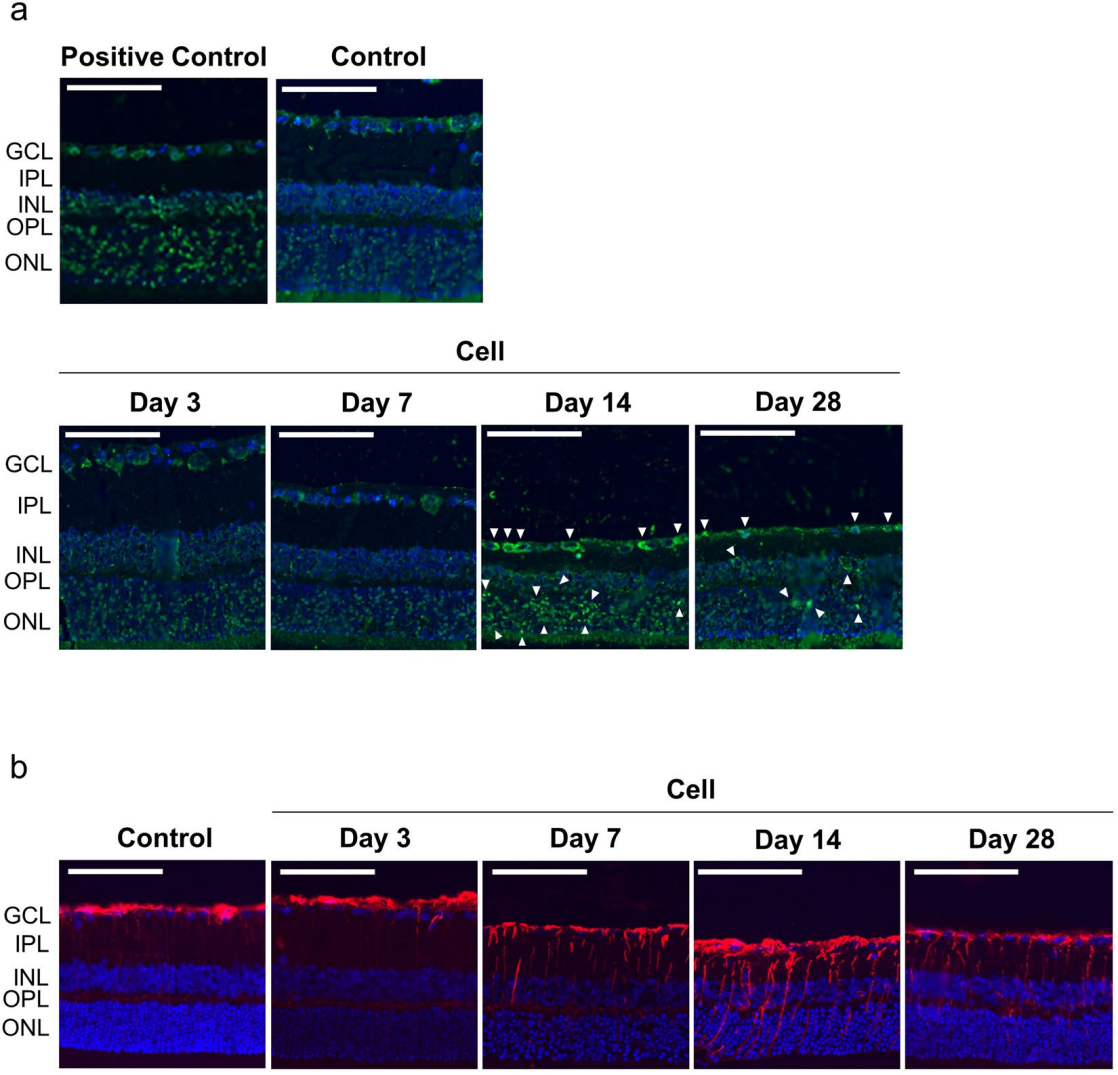
TUNEL and immunostaining for GFAP in retinal cells. Representative images of TUNEL-stained and GFAP-immunostained retinal sections 2 mm away from the optic nerve head. (**a**) TUNEL positivity was observed in the GCL, INL, and ONL of retinae in the injected eyes 14 days after cell injection (arrow heads) but decreased by 28 days after the injection. Positive controls were treated with DNase I to induce DNA fragmentation. Nuclei of cells were stained with DAPI (blue). Scale bars represent 100 µm. (**b**) GFAP positivity (red) was only observed in the GCL in control eyes. However, GFAP expression gradually extended throughout the retina from the GCL to ONL starting on the 7th day after cell injection, and it reached a peak 14 days after the injection. GFAP expression decreased by the 28th day after the injection. Nuclei of the cells were stained with DAPI (blue). Scale bars represent 100 µm. GCL, ganglion cell layer; IPL, inner plexiform layer; INL, inner nuclear layer; OPL, outer plexiform layer; ONL, outer nuclear layer.

TUNEL assays were performed to investigate how RGC loss was induced after cell injection. TUNEL-positive cells were observed in the ganglion cell layer (GCL), INL, and ONL 14 days after cell injection in the Under-50 group (Fig. 6a). However, the number of TUNEL-positive cells decreased by 28 days after cell injection.

### Immunostaining for glial fibrillary acidic protein (GFAP) in retinal cells

To detect the activation of glial cells, a typical sign of glaucomatous neuropathic damage, we evaluated the induction of GFAP expression in retinae. Positive staining for GFAP was restricted to the GCL, co-localizing with astrocytes, in control eyes (Fig. 6b), but GFAP was expressed in Müller glial cells throughout the GCL, IPL, and INL in the injected eyes of the Under-50 group 7 days after cell injection and was present in all retinal layers, reaching its highest level, 14 days after cell injection. GFAP expression was attenuated 28 days after cell injection.

## Discussion

COH models have been developed in several animal species, and rat COH models, in particular, are widely studied because rats are easy to use in large numbers of experiments in preclinical settings. The techniques used in establishing these models include episcleral vein cauterization^20^ and ligation^13,21^, trabecular meshwork laser photocoagulation^22,23^, episcleral and limbal vein laser photocoagulation^23^, hypertonic saline injection into the episcleral vein^24^ and intracameral injection of microbeads^25^. In some cases, however, it is technically difficult to induce IOP elevation. Furthermore, few reports have demonstrated the pathological changes in their models sequentially. Thus, we developed a new COH model and performed a time-course analysis using the model.

In this study, we developed a rat COH model using a method that we previously established in ferrets^18^. The idea of intracameral injection of conjunctival fibroblasts is based on the clinical occurrence of epithelial downgrowth into the anterior chamber, which has often been observed during invasive ocular surgery with a wide corneal incision. Intracameral injection of conjunctival fibroblasts into the rat anterior chamber induced significant IOP elevation, relative to that in control eyes, 3 days after cell injection. The peak IOP was reached on the 7th day, and IOP remained at higher-than-normal levels until 28 days after cell injection. In our model, cell injection succeeded in inducing high IOP in 70.9% of the cell-injected eyes. This result shows that long-term IOP elevation could be stably induced in our rat model. In this model, slit-lamp examination revealed cell accumulation on the corneal endothelium and anterior synechiae starting at 3 days after cell injection. These changes finally led to the obstruction of the aqueous outflow, followed by IOP elevation. A longitudinal study is required to confirm the appropriate model for OAG.

Maintaining IOP elevation at a desired level has been one of the problems with existing experimental COH models. IOP elevations of higher than 50 mmHg were observed in 28.2% of the eyes with elevated IOP in this study. It has been reported that abrupt increases in IOP values to higher than 50 mmHg in rats lead to ONH degeneration, followed by a reduction in the retinal blood flow rate and diameter^26–28^. This finding suggests that an IOP higher than 50 mmHg causes ischemic optic neuropathy identified in APAC and unlike COH models that mimic primary open-angle glaucoma (POAG)^29,30^. Therefore, we compared RGC degeneration between the Over-50 group and the Under-50 group (Fig. 5). IOP-dependent RGC loss occurred 14 days after cell injection in both groups. However, 28 days after cell injection, the pattern of RGC loss differed between the two groups. In the Under-50 group, RGC loss increased in an IOP-dependent manner (Fig. 5b). This result indicates that chronic high IOP led to IOP-dependent cell death, mimicking the clinical pathology of POAG. Conversely, in the Over-50 group, severe RGC loss occurred on the 28th day after cell injection without correlation with IOP (Fig. 5b). In clinical APAC, abrupt elevation in IOP elicits ischemic optic neuropathy rather than glaucomatous optic neuropathy with chronic deformation of the lamina cribrosa. This suggests that IOP values exceeding 50 mmHg in our model induced severe RGC loss by ischemic optic neuropathy; these cases can be classified as having APAC. Therefore, the Over-50 group in this model may be useful for developing neuroprotective drugs for APAC-induced optic neuropathy.

In the Under-50 group, characteristic features of glaucoma, such as thinning of the retina and increased ONH cupping, were confirmed. Mechanical compression by IOP elevation leads to the deformation of the lamina cribrosa in humans or the glial lamina in rats, and then compression of retinal nerve fibers results in RGC dysfunction in glaucoma^6,31^. The histological and morphological changes leading to the development of retinal thinning and increased ONH cupping were also observed in our model (Fig. 2d,e). These results support the claim that optic neuropathy is caused by ONH compression following IOP elevation in our model, similar to the changes in POAG. Meanwhile, eyeballs with elevated IOP were enlarged, and it was predicted that the entire posterior retina was exposed to high pressure. Thus, not only did optic neuropathy result from ONH compression but retinal degeneration was also induced by mechanical compression of the retinae in our model. Longitudinally, this eyeball expansion might continue because flexibility of the sclera in young (9-week-old) rats could predispose their eyeballs to expansion by pressure^32,33^. Moreover, this global expansion may compensate for the high IOP-induced focal stress on ONH. Thus, older rats may be ideal as glaucoma models with severe ONH deformation because of lower induction of eyeball expansion, thereby exposing the ONH to higher pressure.

TUNEL-positive cells were identified in the GCL, INL and ONL 14 days after cell injection. Kerrigan *et al*. have reported that the retinae of glaucoma patients contain an increased number of TUNEL-positive cells^34^, and our results corroborate these clinical observations. Glial activation, a marker of neurological disorder characterized by increased GFAP expression, is also observed in the retinae of glaucoma patients and various experimental COH models^21,35–37^. In this study, GFAP expression was limited to the GCL, co-localizing with astrocytes, in control eyes but was observed in Müller glial cells throughout the GCL extending to the ONL from the 7th day to a peak expression on the 14th day after cell injection. These results suggest that our model exhibits the pathological changes that occur in glaucoma and that the neurological disorder in our model progresses from approximately the 7th day to the 14th day after cell injection.

There are several limitations in this study. First, we only examined changes in IOP and morphology until 28 days after cell injection. Analysis of the duration of elevated IOP should be extended beyond a month after cell injection. Second, while long-lasting IOP elevation was achieved at a high rate, IOP values were difficult to control. It should be possible to control the degree of IOP elevation by varying the cell injection protocol. Third, the main disadvantage of our model is that the view of the posterior chamber of the eye was continuously obstructed by cell accumulation and iris adhesion at the front of the lens. We need to improve our method for producing occlusion of the angle by restricting cell accumulation to the peripheral area of the anterior chamber. Fourth, the possibility that an inflammatory response to injected cells occurred in the anterior segment cannot be excluded. However, it is possible to select animals with limited anterior damage. Finally, as mentioned in a recent review of glaucoma animal models^38^, rodents have only a glial lamina rather than a structured lamina cribrosa. Thus, humans or primates may exhibit a different pathology of axon damage and pattern of progression. Nevertheless, in contrast to models with a structured lamina cribrosa, our rat model may still be used to clarify the initial site or mechanism of axon damage.

In this study, we developed a reproducible rat glaucoma model with sustained IOP elevation using intracameral injection of conjunctival fibroblasts. In addition, our model was classified into two groups based on the cut-off value of IOP, and each group displayed different types of RGC loss. One type of RGC loss represented chronic optic neuropathy, which developed after an increase in ONH cupping and RGC dysfunction and mimicked the clinical pathology of POAG. The other type was severe RGC loss, mimicking APAC. Our model has great potential for further investigation of the pathogenic mechanisms of glaucoma and may be useful in the development of IOP-lowering drugs and neuroprotective drugs.

## Methods

### Animals

All animals used in this study were treated in accordance with the ARVO Statement for the Use of Animals in Ophthalmic and Vision Research. The protocol was approved by the Institutional Animal Care and Use Committee of Research Laboratories at Senju Pharmaceutical Co., Ltd. (No. 20150116-01). Nine-week-old male Sprague-Dawley rats were obtained from Charles River Japan (Yokohama, Japan), housed at 23 °C with a 12-hour light/dark cycle, and fed ad libitum.

### Preparation of conjunctival fibroblasts

Conjunctival tissues were excised from five rat eyes and incubated in Dulbecco’s Minimum Essential Medium (DMEM, Invitrogen, Carlsbad, CA, USA) with 0.2% dispase^®^ II (Roche, Basel, Switzerland) for 1 hour at 37 °C. Epithelial cells were mechanically removed, and the remaining tissue was minced and digested with 0.1% collagenase A (Roche) overnight at 37 °C. Conjunctival fibroblasts in collagenase solution were then re-suspended in DMEM supplemented with 10% fetal bovine serum (FBS) and 1% penicillin/streptomycin (all from Invitrogen) and cultured under standard conditions (humidified atmosphere of 5% CO_2_ at 37 °C). Cultured conjunctival fibroblasts were repeatedly cultured after achieving 70%–90% confluence.

### Intracameral injection of conjunctival fibroblasts

After achieving approximately 90% confluence, conjunctival fibroblasts were dissociated with TrypLE™ Express (Invitrogen) and suspended in DMEM supplemented with 10% FBS and 1% penicillin/streptomycin (1.5 × 10^7^ cells/mL). After topical administration of 0.5% tropicamide and 0.5% phenylephrine mixed eye drops (Mydrin-P, Santen Pharmaceuticals, Osaka, Japan), animals were anesthetized with intraperitoneal ketamine (100 mg/kg) and xylazine (10 mg/kg). Then, topical 0.4% oxybuprocaine (Benoxyl, Santen Pharmaceuticals) diluted 10-fold and 0.3% gatifloxacin (Gatifro, Senju Pharmaceutical, Osaka, Japan) were applied to the corneal surface. Five microliters of cell suspension was injected slowly over 2–3 minutes into the anterior chamber of one eye with a 34-gauge needle (n = 55), and the contralateral eye was left untreated. In addition, 5 µL of culture medium was unilaterally injected into the anterior chamber of one eye in other rats as a control (n = 31). The contralateral eyes of animals in both groups were untreated and regarded as normal eyes. After injection, the eyes were treated with an ointment containing 0.3% ofloxacin (Tarivid, Santen Pharmaceuticals), and animals were given an intraperitoneal injection of 1 mg/kg atipamezole (Antisedan, Nippon Zenyaku Kogyo, Fukushima, Japan) to facilitate recovery from anesthesia.

### Measurement of intraocular pressure (IOP)

After inducing anesthesia with sevoflurane, IOP was measured using a rebound TonoLab^®^ (iCare, Helsinki, Finland) 1 day before (baseline) and 3, 7, 14, 21 and 28 days after intracameral injection. IOP measurements were started 5 minutes after the induction of anesthesia and completed within 5 minutes to circumvent the effect of anesthesia on IOP. All measurements were conducted between 9:00 and 12:00. To avoid drying of the cornea, we administered 0.1% hyaluronic acid (Tearbalance, Senju Pharmaceutical) during the maintenance of anesthesia. Three IOP values were obtained from each eye, and the daily mean IOP values for each eye were calculated. The area under the curve (AUC) of IOP change from baseline in each injected eye was calculated 3, 7, 14, and 28 days after cell injection.

### Slit-lamp observation of the anterior chamber

The anterior segments of eyes were examined with a slit lamp (Topcon, Tokyo, Japan) at the same time as the IOP measurements were made.

### Tissue preparation

Animals were deeply anaesthetized by inhalation of air-saturated isoflurane and perfused with cold saline followed by 4% paraformaldehyde (PFA) on the 3rd, 7th, 14th or 28th day after intracameral injection. Eyes were enucleated and post-fixed with 4% PFA.

### Histological analysis

Three-µm paraffin-embedded sections and 8-µm cryosections were prepared and stained with hematoxylin and eosin. Changes in the anterior segment, retinal thickness, and ONH cupping were examined using the bright-field mode of a fluorescence microscope.

### RGC counting

After overnight fixation, retinae were flat-mounted and then incubated with a primary antibody against BRN3A (Millipore, Billerica, MA, USA), a transcription factor specifically expressed in the nuclei of ganglion cells. Then, retinae were incubated with a secondary antibody conjugated to Alexa Fluor^®^ 488 (Invitrogen). Regions that were located 1 mm and 2 mm away from the ONH in each of four quadrants were photographed with a fluorescence microscope (Keyence, Osaka, Japan). BRN3A-positive RGCs were counted in fields of 0.4 mm^2^ using an image visualization software (Image-Pro Plus, Nippon Roper, Tokyo, Japan). The counts of RGCs in 8 areas of each eye were averaged, and the mean numbers of RGCs for each group were calculated. The rate of RGC loss was defined as the percentage decrease in the number of cells in the cell-injected eye relative to the number of cells in the control (culture medium-injected) eye.

### TUNEL assay

Terminal deoxynucleotidyl transferase dUTP nick end labeling (TUNEL) staining of cryosections was carried out using the Click-iT^®^ TUNEL Alexa Fluor^®^ 488 Imaging Assay (Invitrogen) to detect DNA degradation that occurs during apoptosis *in situ*. Sections were embedded with Vectashield^®^ Mounting Media with 4′,6-diamidino-2-phenylindole (DAPI) (Vector Laboratories, Burlingame, CA, USA) and examined under a fluorescence microscope. Positive controls were prepared by treating sections with DNase I before TUNEL to detect DNA fragmentation.

### Immunohistochemical analysis

Eight-µm-thick cryosections were cut and immersed in 10 mM phosphate-buffered saline (PBS). The sections were incubated with a blocking solution containing 5% bovine serum albumin/0.1% Triton X-100/10 mM PBS at room temperature for 20 minutes to eliminate non-specific binding, incubated with primary antibodies against glial fibrillary acid protein (GFAP, Dako, Glostrup, Denmark) at 4 °C overnight, and washed with PBS. Then, they were incubated with secondary antibodies conjugated to Alexa Fluor^®^ 546 (Invitrogen) at room temperature for 1 hour and embedded with Vectashield^®^ Mounting Media with DAPI (Vector Laboratories). Immunofluorescence images were acquired using fluorescence microscopy.

### Statistical analysis

The IOP values and RGC counts are presented as the mean ± standard deviation (S.D.). The differences in IOP values and RGC counts between the injected eyes and the control eyes were statistically analyzed with two-tailed Student’s *t*-tests of data from each day after cell injection. Linear regressions with Pearson’s correlation coefficients were performed between normalized RGC numbers and AUCs of the differential IOP from baseline in cell-injected eyes for the Under-50 and Over-50 groups. p < 0.05 was considered statistically significant.

## Data Availability

The datasets generated and/or analyzed during the current study are available from the corresponding author upon reasonable request.
